# Reproducing asymmetrical spine shape fluctuations in a model of actin dynamics predicts self-organized criticality

**DOI:** 10.1038/s41598-021-83331-9

**Published:** 2021-02-17

**Authors:** Mayte Bonilla-Quintana, Florentin Wörgötter, Elisa D’Este, Christian Tetzlaff, Michael Fauth

**Affiliations:** 1 Department for Computational Neuroscience, University of Göttingen, 37077 Göttingen, Germany; 2 Bernstein Center for Computational Neuroscience, University of Göttingen, 37077 Göttingen, Germany; 3 Max-Planck-Institute for Medical Research, Optical Microscopy Facility, 69120 Heidelberg, Germany

**Keywords:** Biophysical models, Computational models

## Abstract

Dendritic spines change their size and shape spontaneously, but the function of this remains unclear. Here, we address this in a biophysical model of spine fluctuations, which reproduces experimentally measured spine fluctuations. For this, we characterize size- and shape fluctuations from confocal microscopy image sequences using autoregressive models and a new set of shape descriptors derived from circular statistics. Using the biophysical model, we extrapolate into longer temporal intervals and find the presence of 1/*f* noise. When investigating its origins, the model predicts that the actin dynamics underlying shape fluctuations self-organizes into a critical state, which creates a fine balance between static actin filaments and free monomers. In a comparison against a non-critical model, we show that this state facilitates spine enlargement, which happens after LTP induction. Thus, ongoing spine shape fluctuations might be necessary to react quickly to plasticity events.

## Introduction

Dendritic spines are small protrusions of dendrites where the postsynaptic part of most excitatory synapses is located. It is well known that size and shape changes of these spines are correlated with changes of the strength of excitatory synaptic connections^1^. However, Fischer et al.^2^ observed that the shape of dendritic spines also varies spontaneously if spines are at the minimum level of activity, which is required to maintain their functions. Interestingly, nearly all spines in these recordings survived and their volume and density stayed mostly constant. Similar spine variations were measured in different types of cells and under different experimental settings^3–5^.

Rapid changes of dendritic spine shapes are related to actin dynamics^2,3^. Actin is a globular protein that assembles into filaments, which are polar structures that continuously undergo a treadmilling process. In this process, actin monomers are polymerized at the (+) end (barbed end) of the actin filaments, while these filaments are depolymerized at the (−) end. Actin polymerization generates a force that moves the cell membrane forward^6^. Moreover, blockage of actin polymerization hinders spine motility^2,3^. Honkura et al.^7^ observed filamentous actin within a single spine and found that a dynamic pool of actin, which has a fast treadmilling velocity and is mainly localized at the tip of the spine, can account for fast spine motility. However, Frost et al.^8^ observed that actin treadmilling velocity is elevated not only at the spine tip, but also in discrete and well-separated foci.

There are numerous theoretical and experimental studies of dendritic spine size changes. However, these studies mostly consider spine development^9,10^, the effects of long-term potentiation^11^, or spine volume fluctuations but at longer time scale of tens of minutes^12^ up to days^13^. Recently, Bonilla-Quintana et al.^14^ proposed an actin based model that mimics rapid spine motility of around 100 ms. However, to date it remains unclear whether these rapid shape fluctuations have a possible function and, if they have, which function they fulfill^15^.

Here, we use a biologically realistic theoretical model to study rapid, spontaneous shape fluctuations of dendritic spines generated by the dynamics of actin, observed by Fisher et al.^2^ and others^3–5^. For this, we first demonstrate that the model dynamics matches experimental time-lapse data previously published in Mikhaylova et al.^16^ as well as our new data. The model allows us to predict spine characteristics for periods of time that exceed those currently accessible experimentally. Analyzing such long time intervals, we find that the spine fluctuations exhibit 1/*f* noise, which can be explained by the actin polymerization dynamics that occurs in bursts (“avalanches”) from specific polymerization foci. The model further predicts that these polymerization dynamics self-organizes into a critical state^17^. We demonstrate that this type of self-organized criticality underlying the spine fluctuations would be functionally advantageous, as it enables the spine to react faster and more strongly to changes in the molecular dynamics which occur, for example, during structural long-term potentiation (sLTP) where spine enlargement results from structural rearrangements of actin filaments upon activation of NMDA receptors^18^.

## Results

### Fundamental behavior of the model

**Figure 1:**
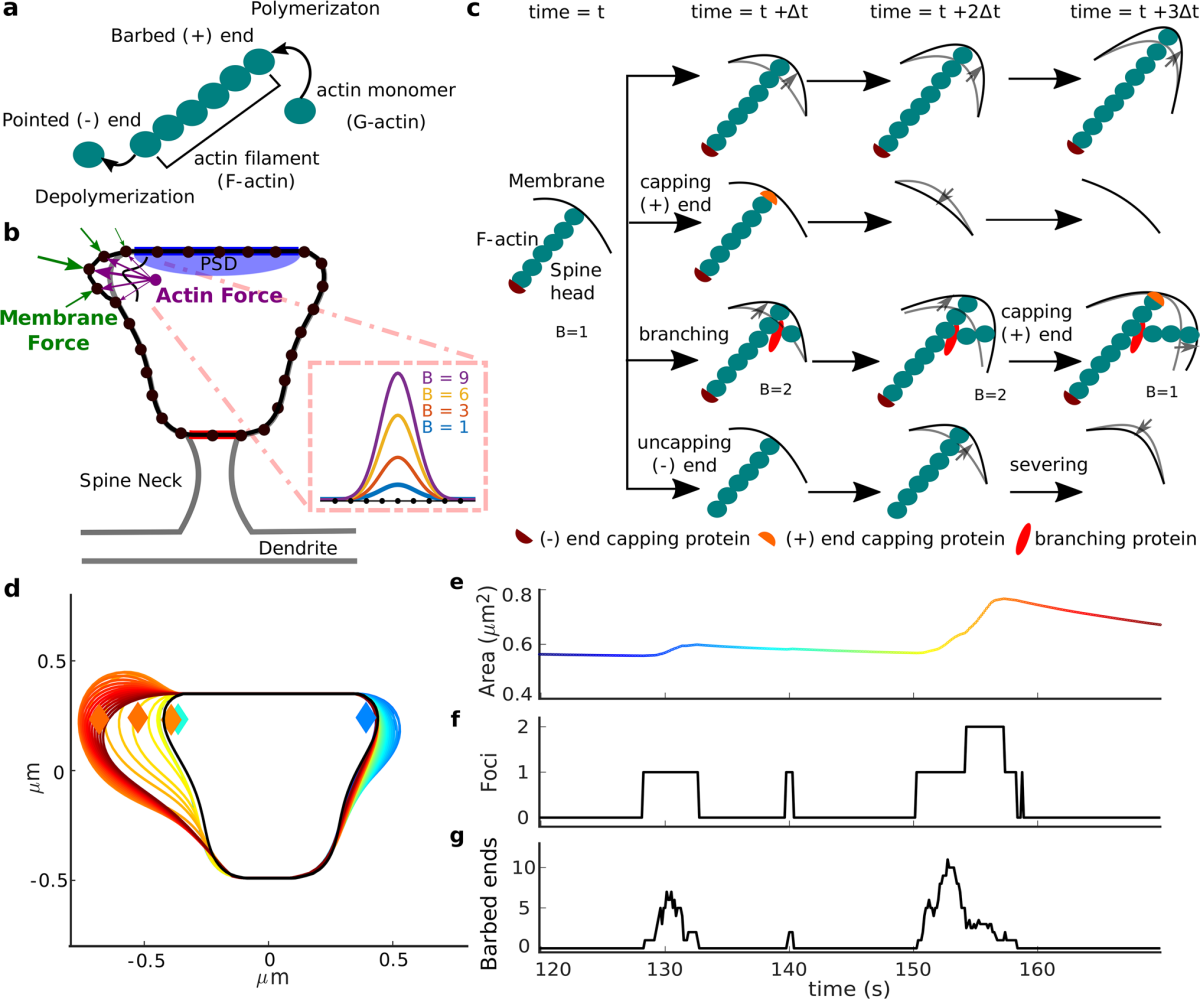
Description of the theoretical model. The mechanisms that displace and change PSD and spine neck are not shown in this figure. (**a**) Descripiton of the actin treadmilling process. (**b**) Characterization of the force interactions in a dendritic spine head. Black dots are the mesh approximation for the membrane. Arrows represent the forces exerted by the membrane and actin polymerization, in green and purple, respectively. The purple dot signals the nucleation location of an actin polymerization focus. The vertices representing the PSD and neck are highlighted in blue and red. The inset shows the Gaussian kernels for different number of barbed ends *B*. (**c**) Filament events for 4 time-steps (columns), each row shows a different scenario. Changes in membrane are denoted by gray arrows. At each time-step, all filaments with uncapped (+) end, barbed end *B*, undergo monomer addition and the membrane is expanded, as shown in the first row. However, monomer addition at the (+) end stops when it is capped by a capping protein (second row). Thereupon, the actin filaments do not polymerize and hence, the membrane eventually recovers its initial shape because there is no force pushing it forward. Besides these events, actin filaments with barbed ends can branch, and thus, $B \rightarrow {B+1}$ (third row). Also, the actin filament capped (−) ends can be uncapped by removing the capping protein. Uncapped minus ends are eventually severed, which eliminates the filament and thus, the membrane is no longer deformed (fourth row). (**d**) Spine shapes arising from model simulation, color-code according to time as in (**e**). Black line represents the “resting” shape to which the spine shrinks in the absence of actin force (see “Methods”). Diamonds represent actin nucleation locations, initiated at different times (color-coded). (**e**) Area evolution of the spine, color-coded from initial (in dark blue) to final time. (**f**) Number of polymerization foci during this period. (**g**) Number of uncapped (+) ends, at the polymerization foci over time. See “Methods” for further details.

To investigate the influence of actin dynamics on the spine shape fluctuations, we adapted and extended our previously published model^14^, in which asymmetric shape fluctuations emerge from an imbalance between an expanding force generated by actin polymerization and a force generated by the lipid membrane, that counteracts shape deformations (Fig. 1b). The model is based on the assumption that actin polymerization, relevant for shape changes, mainly occurs at distinct polymerization foci^8^. The model only considers actin filaments polymerizing at fast rate touching the membrane. Because of the small number of total actin filaments in the spine head (around 100 filaments^10^), and that only few of these are relevant for producing a force against the membrane, a Monte Carlo approach is used to describe the actin network in each polymerization focus.

In each of these foci, actin continuously undergoes a treadmilling process in which monomers (G-actin) are polymerized at the (+) end of the filaments (also called the “barbed end”), whilst G-actin is depolymerized or severed at the pointed (−) end (Fig. 1a), where in case of dominance of polymerization an expanding force is generated.

In addition to the treadmilling process, other events can occur in the filaments that affect the number of barbed ends at a polymerization focus, namely, barbed ends can branch and form new filaments with barbed end and capped (−) end, or barbed ends can be capped, which blocks polymerization. Additionally, capped minus ends can be uncapped, and filaments with uncapped (−) ends can be severed (see Fig. 1c). Importantly, the branching probability depends on the membrane force at that location and on the number of barbed ends. Consequently, it decreases when the force generated by actin pushes the membrane extensively increasing the force generated by the membrane, or when there is an increased number of barbed ends, and hence, it acts like a negative feedback mechanism (Supplementary Information Fig. S1, see “Methods” for more details of the branching rate).

Due to these processes, the number of barbed ends at each focus fluctuates until a focus becomes extinct and vanishes within seconds. Meanwhile, however, new actin polymerization foci are generated with a given probability, proportional to the nucleation rate, at a randomly chosen location near to the postsynaptic density (PSD, purple dot in Fig. 1b) that push the membrane in a chosen direction (thick purple arrow in Fig. 1b). The number of barbed ends is counted at each time-step to calculate the force generated by actin polymerization. Instead considering single actin filaments, we implemented a spatial Gaussian kernel, proportional to the number of barbed ends, to calculate this force (see magnification in Fig. 1b). In addition to these basic model properties, we also used mechanisms that allow slow and small displacements and changes of PSD as well as spine neck (see “Methods”).

In the model, shape fluctuations mainly arise from the continuous nucleation and disappearance of actin polymerization foci at different locations^14^ (Fig. 1e) . The membrane expands and the spine area increases when the barbed ends at a focus are polymerizing (Fig. 1d–g). Due to the stochasticity of the F-actin processes, at some point in time, the barbed ends are depleted and the focus extinguishes. Thereupon, the polymerizing force vanishes and the membrane shrinks (Fig. 1d), decreasing the spine area. In the current study, this model was modified using observations from experimental data and exploited to make long-term predictions of spine behavior, not yet possible with experiments.

### Quantification of experimental spine shape fluctuations and matching model

To investigate if our model can reproduce the spine size and shape fluctuations from experimental data, we first described their statistical properties. For this, we re-analysed imaging data from primary mouse neurons at 12 days in vitro (DIV12) previously published in Mikhaylova et al.^16^. These neurons were transfected with mRuby2, as cell fill, and GFP-actin, as actin marker, and imaged every 10 s for 5 min, which provides a sufficient temporal resolution to study spine shape fluctuations. Yet, this high imaging frequency also prevents recording for long time periods due to experimental limitations. To test if our description of spine shape is independent of the data set and if it changes when using many samples from a single spine, instead of considering a population, we analyzed also longer recordings consisting of 100 frames sampled every $\approx 9.6$ s from hippocampal neurons (DIV19, labelled with DiO, see “Methods”).

**Figure 2:**
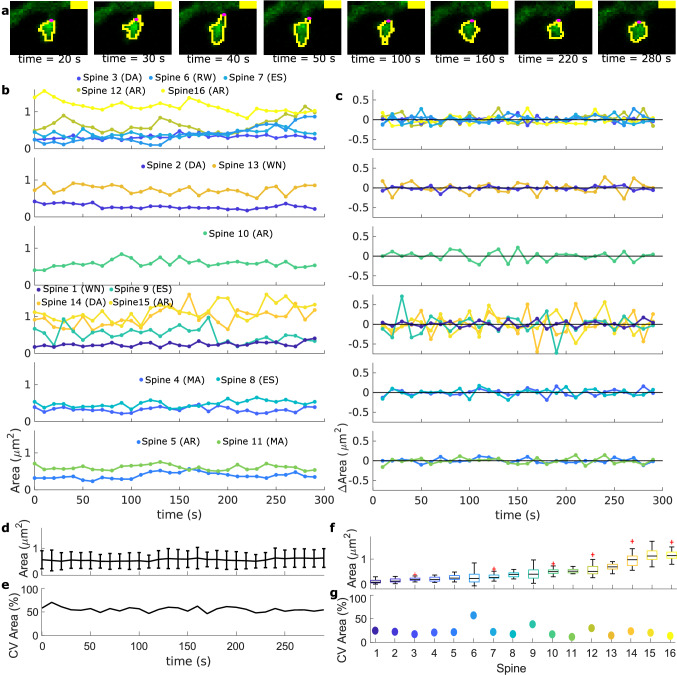
Spine area evolution over time from a population data set. (**a**) Images taken from time-lapse data of a neuron transfected with GFP-actin (green). The yellow polygon represents the analysed ROI and the magenta circle corresponds to the spine neck center. Yellow bar indicates 1$\mu$m. (**b**) Area evolution for spines in different stacks (top to bottom). Spines are color-coded and numbered from small (dark blue) to large (yellow). Abbreviations correspond to the best fit of an ARIMA model (see Supplementary Information, Table S1). (**c**) Difference of area, $\Delta \text {Area}$, from spines in (**b**) over time. (**d**) Mean ± standard deviation (std) of the area of the analysed spines at each time-frame. (**e**) Coefficient of variation (CV) from (**d**). (**f**) Boxplots of spine area evolution for each spine. (**g**) CV from (**f**).

First, we assessed the nature of the fluctuations in spine *size*. To extract the spine shape, we used Fiji^19^ to analyse the images and traced a region of interest (ROI) around the heads of the spines for each time frame (yellow polygon in Fig. 2a, see “Methods” and Supplementary Information Fig. S2 for full details). We calculated the area at each time-frame and to quantitatively describe its tendency over time, we performed a time series analysis using autoregressive integrated moving average (ARIMA) models (see details in “Methods”). Thus, possible tendencies in area evolution express themselves through which ARIMA model best fits the data. In particular, the best fitting model reveals whether the fluctuations rather correspond to white (shot) noise (WN=ARIMA(0,0,0)), a random walk (RW=ARIMA(0,1,0)) or whether they exhibit the tendency to return to a stationary (AR=ARIMA(1,0,0)) or moving (ES=ARIMA(0,1,1)) mean value. Secondly, we developed a set of descriptors that characterize the asymmetry in spine *shape*.

**Figure 3:**
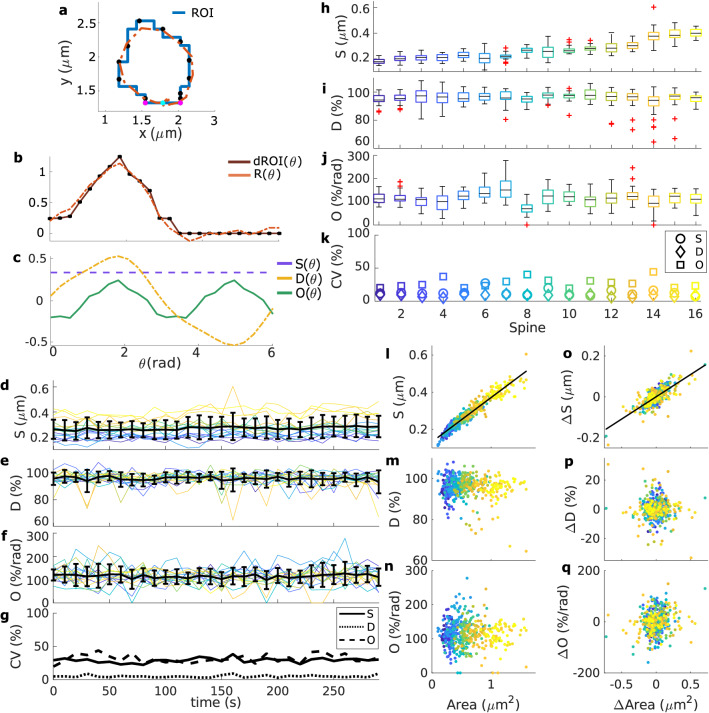
Shape descriptors of spines from a population data set. (**a**) Blue line: ROI extracted from data corresponding to the spine in Fig. 2a at time = 280 s. Black dots correspond to the sampled points of the ROI and the orange dotted line to the approximation function $R(\theta )$. Magenta dot denotes the spine neck center and cyan dots the neck limits. (**b**) Plot of the distance between the neck and sample points $\text {dROI}(\theta )$ (brown line) and $R(\theta )$ (orange line) over the sampling angles $\theta _i$ (in radians). (**c**) The three parts of $R(\theta )$ corresponding to the general size of the spine $S(\theta )$, the directional selectivity $D(\theta )$, and orientational selectivity $O(\theta )$ (see “Methods” for details). (**d–f**) Shape descriptors, *S*, *D*, and *O*, evolution over time for different spines. (**g**) Coefficient of variance corresponding to (**d**–**f**). (**h–j**) Box plot of shape descriptors for the analyzed spines, color-coded as in Fig. 2. (**k**) Coefficient of variance of the shape descriptor for each spine. (**l–n**) Spine area against shape descriptors *S*, *D* and *O*, respectively. Each dot represents the corresponding value at a time-frame for a certain spine (color-coded). Black line is a linear least-squares fit. Only *S* correlates significantly with the area (p-value $<\, 0.01$; Pearson’s linear correlation coefficient cc$=0.96$). (**o–q**) Same as (**l**–**m**) but using the time differences of the shape descriptors. $\Delta S$ correlates with $\Delta$Area (cc$= 0.77$).

Spine shapes are highly asymmetric, and therefore, common shape factors fail to describe them. Therefore, we propose here new shape descriptors, based on concepts from circular statistics^20^, that detect asymmetries, such as changes in spine elongation or tilting. In short, these descriptors measure the direction- *D* and orientation *O* selectivity of the spine respect to the neck center, and the overall distance from the spine neck *S* (Fig. 3a,b). See “Methods” for details of how to compute this and also Supplementary Information (Fig. S3) for more examples.

#### Analysis of experimental data

First, we examined the spine area evolution from the first data set, calculated from the extracted ROIs (yellow polygons in Fig. 2a). In the same stack (i.e., image set of the same frame taken at different times), spines with different sizes and fluctuation behavior were observed (Fig. 2b,c). However, we ruled out any drifts in the data by verifying that the average (Fig. 2d) and the coefficient of variability (Fig. 2e) of the spine sizes over the population were conserved at each frame.

The average area of the spines spreads over a continuous range (Fig. 2f) and the variability does not correlate to spine size. To quantitatively test the latter, we calculated the coefficient of variance (CV) for each spine time evolution (see Fig. 2g). In sum, we observed that spines behave differently and without any correlation to their size. For example, big spines exhibit large (Spine 12), but also small (Spine 16) area fluctuations that can be either fast (Spine 15) or slow (Spines 12, 16). And the same is true for small spines (compare Spine 6 with Spines 1, 5).

When examining the spine area differences between successive time frames, given by $\Delta \text {Area}(t_i) = \text {Area}(t_i) - \text {Area}(t_{i-1})$ (Fig. 2c), we noted a tendency to counteract a positive change in area by a negative change. Hence, the spine area tends to fluctuate around a mean. Such behavior was verified by the ARIMA fittings. We obtained that the area of 5 spines (31.25%, see Supplementary Information, Table S1), best fitted by an ARIMA(1,0,0), tends to return to a constant area mean value, either rapidly (Spines 10, 15) or slowly (Spines 5, 12, 16). Taken together with another 3 spines that fluctuate around slow moving mean values, there is a clear tendency towards fluctuations that have a drive to return towards a mean value. Note that although this was the most common tendency, some spines show qualitatively different behavior (see Supplementary Information, Table S1).

Next, the shape descriptors were calculated for each spine of the first data set at every time-frame. As shown in Fig. 3d–f, the quantities fluctuate over time. Similar to our observation on spine size fluctuations, also here we did not observe any trends or drifts (Fig. 3g). Instead, the descriptors fluctuate around a mean value with small changes in their variability. However, we also noted that these mean values and the coefficient of variation of shape fluctuations can be very different for different spines (Fig. 3h–k). Moreover, the shape descriptors also seem to alternate between positive and negative changes; however, an analysis using ARIMA models (see Supplementary Information, Table S1) shows that the fluctuations in orientational and directional selectivity mostly behave like white noise (50% of the spines), indicating that changes in *D* and *O* are not correlated in time.

Further examination shows that only *S* significantly correlates to the spine area and that changes in area are only strongly correlated to those in *S* (Fig. 3l,o). Such correlations are expected since *S* accounts for the average distance from the spine neck to the membrane. Because *D* and *O* and their differences are not strongly correlated to the spine area and $\Delta \text {Area}$, respectively (Fig. 3m–n,p–q), we concluded that size fluctuations are independent to those in shape, in line with previous experimental results using different measures^2^.

**Figure 4:**
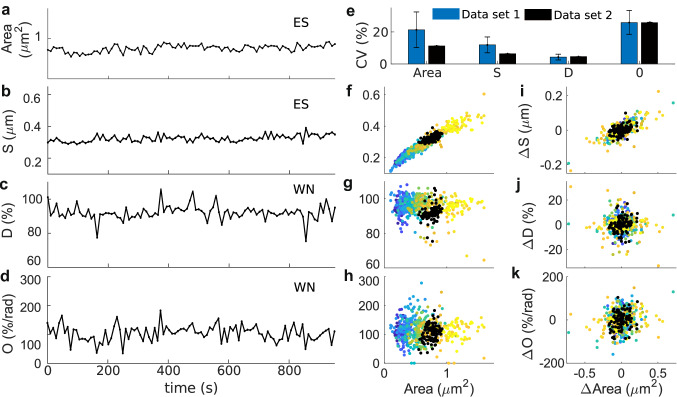
Comparison between different experimental data sets. Here the data set 1 corresponds to that of Fig. 3, and data set 2 to a longer recording. (**a–d**) Area and shape descriptors (*S*, *D*, and *O*) evolution over time for a spine belonging to data set 2. Abbreviations correspond to the best fit of an ARIMA model (see Supplementary Information, Table S1) (**e**), median ± standard deviation of the coefficient of variability (CV) for shape descriptors calculated for each spine in data set 1 (blue) and CV for the spine from data set 2 (black). (**f–h**) Spine area against shape descriptors *S*, *D* and *O*. Each dot represent a measure of shape at a sampled time frame. There are 16 spines sampled every 10 s for 5 min corresponding to data set 1, color-coded as in Fig. 3 and 100 frames from data set 2 sampled every  9.6 s (black). (**i–k**) Changes in area against changes in shape descriptors from the data in (**f**–**h**).

To test whether these observations are independent of the data set, we analyzed long-term data. Figure 4a–d shows that this data behaves similarly: the spine area and *S* fluctuate around a slow moving average whilst the changes in *D* and *O* correspond to white noise. Moreover, the area and its changes are only significantly correlated to *S* and its changes (cc$=0.5917$ and cc$= 0.3949$, with p-value $< 0.01$ using Student’s *t* distribution, respectively, see Fig. 4f,i). Note that the distribution of the area values of the new data set are comparable to those from single spines in the previous data set (see Fig. 4f,k); hence, there is less variability in the area and *S*, but the variability in *D* and *O* is similar (Fig. 4e). Therefore, it appears that spines recorded with different experimental setups can behave similarly.

#### Model reproduces experimental shape fluctuations

**Figure 5:**
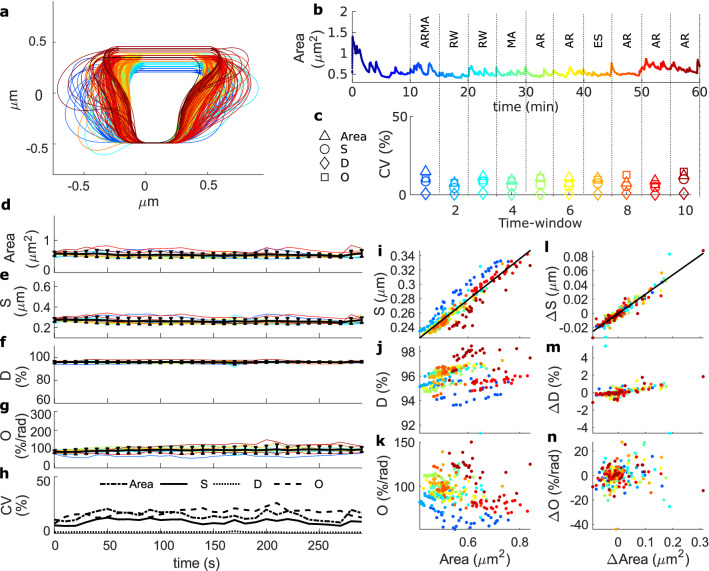
Shape descriptors from the model simulation. (**a**) Spine shapes resulting from the model simulation every 10 s, color-coded from 0 min (dark blue) to 60 min (dark red). (**b**) Spine area evolution over time corresponding to the simulation in (**a**), dotted lines indicate the 5 min windows, the spine number and the best fit of an ARIMA model (abbreviations as in Supplementary Information, Table S1). (**c**) Coefficient of variability of the shape descriptors for each time window. (**d–g**) Shape descriptors over time for the 5 min windows (spines in **a**) of the simulation. As in the experimental data, the values were sampled every 10 s. (**h**) Coefficient of variability of shape descriptor evolution over time. (**i–k**) Spine area against *S*, *D* and *O*. Each dot represent the value for a certain time point and color-code corresponds to the respective 5 min window. Black lines correspond to a linear least-squares fit with a significant Pearson’s correlation coefficient of 0.93 in (**i**) (p-value $< 0.01$ using a Student’s *t* distribution). (**l–n**) Difference in spine area against $\Delta S$, $\Delta D$ and $\Delta O$, respectively. (**l**) Has a significant Pearson’s correlation coefficient of 0.95.

In the next step, we investigated whether our theoretical model reproduces the experimental data. Note that a one-to-one reproduction is not sensible due to the extend of its fluctuations. Instead, we examined whether the model matches the fluctuation statistics of quantities that describe the spine shape. For this, we simulated a spine for a period of 60 min and evaluated time course and fluctuations of the shape descriptors. Figure 5a shows one example of the resulting spine’s shapes. To assess whether or not the diversity of fluctuation characteristics in the experiments emerges as a consequences of short measurement times, we divided one simulation into 5-min time windows with the spine shapes sampled every 10 s (Fig. 5b) and calculated the area and shape descriptors (Fig. 5d–g).

Interestingly, when determining the best-fitting ARIMA models to characterize the fluctuations, we find a similar variety of best-fitting models, although all time-window stem from the same spine. Again, for the spine size, returning to a constant mean is the most common trend (40% of the time windows). Thus, the diversity of best-fitting models is reproduced by the model, if analyzed in the same way as the experimental data, and thus, likely an artifact from short measurement times.

Also the shape descriptors’ time courses are different for each 5-min time window of the simulation (Fig. 5c). Note that this diversity is the result of sustained fluctuations over time (Fig. 5d–g), rather than of a large single change. Hence, the variability over time is maintained (Fig. 5h). We furthermore assessed whether the correlations of area with shape descriptors is similar as in experiments and found that, also for the model, only the spine area and its changes are significantly correlated to those in *S* (Fig. 5i–n).

**Figure 6:**
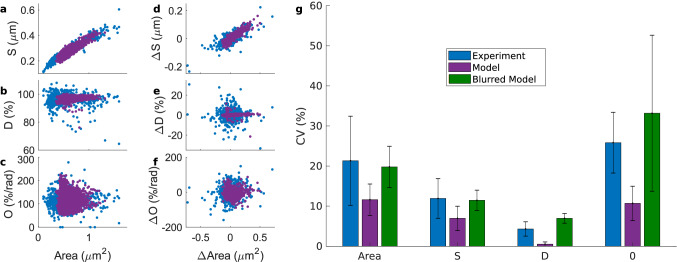
Comparison between experimental data and model. (**a–c**) Spine area against shape descriptors *S*, *D* and *O*. Each dot represents a data point at a sampled time frame. There are 16 spines sampled every 10 s for 5 min corresponding to experimental data from Fig. 3 (in blue) and frames for the model (purple) correspond to 10 simulations in which shapes are sampled every 10 s from minute 10 to minute 60. (**d–f**) Changes in shape descriptors from the data in (**a**–**c**) against changes in area. (**g**) Median ± standard deviation of the coefficient of variability for shape descriptors calculated for each spine, in experimental data or in the model. Green bar corresponds to the CV’s of the model sampled shapes, but blurred.

To improve on the analysis, we ran ten 60-min simulations. Figure 6 shows that the model reproduces the same shape characteristics as the experimental data. The difference between the shape descriptors’ CVs of the model and the experimental data can be due to inaccuracies in the extracted ROIs. Thus, instead of calculating the descriptors from the original simulation spine images, we calculated them from modified model images that better resemble confocal microscopy ones. Such images, obtained by randomly allocating fluorophores inside the modeled spines and blurring them, were analyzed like experimental data on Fiji. In the Supplementary Information (Figs. S4 and S5, and Table S2) we provide more controls of this kind showing that the variability of the shape descriptors is increased and, this way, matches the higher variability of the experimental data. Therefore, the difference in CVs between experimental data and model is very likely due to inaccuracies in obtaining a ROI from experimental data. These results demonstrate that our theoretical model reproduces the characteristics of spine size and shape fluctuations observed in experiments qualitatively and quantitatively.

### Model predictions

#### Extrapolation to long timescales and the presence of 1/*f* noise

After showing that the model can reproduce experimental data, we used it to investigate fluctuation dynamics on timescales that exceed those currently accessible by experiments, and to interrelate these fluctuations with actin dynamics.

**Figure 7:**
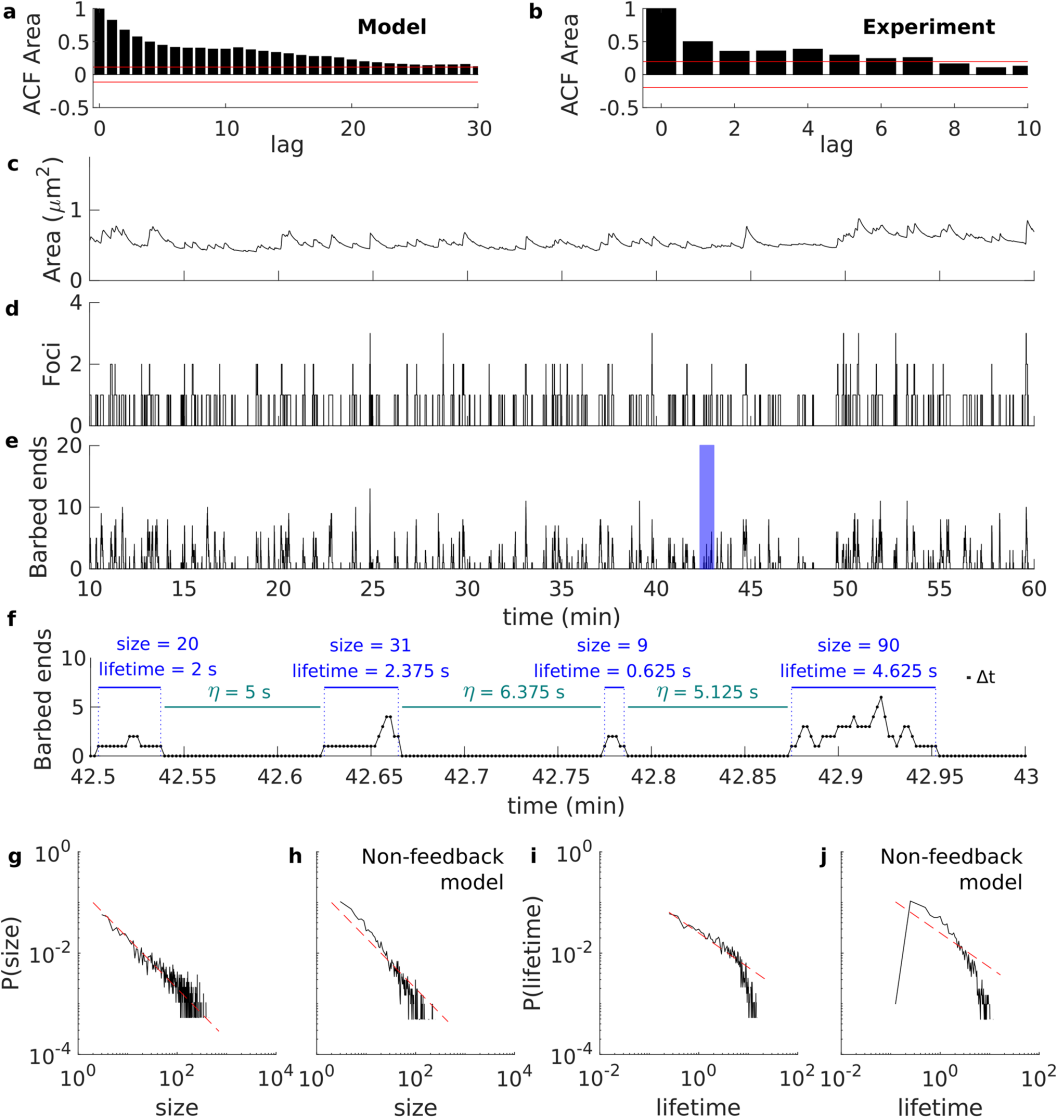
Actin in spines self-organizes into a critical state. (**a**) First lags (10%) of the autocorrelation function of the sampled area from Fig. 5b. Dashed red lines correspond to the 95% confidence bands. (**b**) Same as (**a**) but calculated from the experimental data in Fig. 4a. Spine area (**c**), number of actin polymerization foci (**d**), and total number of barbed ends in the spine (**e**) corresponding to the simulation in Fig. 5. (**f**) Zoom of the blue-shaded interval in (**e**). Each dot indicates the number of barbed ends at each time-step. The size and duration of the avalanches of actin polymerization, as well as the interval between avalanches ($\eta$), is displayed. Log–log plot of the probability of avalanche size (**g**), and lifetime (**i**) corresponding to the data from 10 simulations of the model. Red dotted lines correspond to straight lines with slope of − 1 (**g**) and − 0.68 (**i**), plotted for visualization purposes. (**h**,**j**) Same as in (**g**) and (**i**) but using the simulations of the model without feedback in the branching rate.

As discussed above, the area evolution within every 5-min time window in the model shows different tendencies, even for consecutive windows; however, returning to a constant mean is the most common trend (AR in Fig. 5b), as in the experimental data. In these cases, the area evolution corresponding to the sampled points from Fig. 5b sometimes fluctuates around low values and other times around high values. However, when considering all 60 min of the simulation, there is no sustained increase or decrease of the mean and standard deviation of the area, or any cyclical trend, i.e., the time series is stationary (according to an augmented Dickey-Fuller test with p-value $< 0.01$). Further analysis shows that the autocorrelation function of these sample points follows a slow asymptotic decay (Fig. 7a), which is a feature of 1/*f* noise. This is also observed in the longer experimental recording (Figs. 4a and 7b). The main characteristic of this type of noise is that it arises from a stochastic process with spectral density *SD*(*f*) that follows a power law, i.e., $SD(f)=1/f^\alpha$, with frequency *f* and power law exponent $0.5\lesssim \alpha \lesssim 1.5$.

To further verify the presence of 1/*f* noise in the time series, we used the method developed by Wagenmakers et al.^21^. This method is based on the idea that 1/*f* noise (related to long term correlations) appears between white noise and a random walk (see “Methods” for details). When applying this method to the sampled area trace from Fig. 5b, we verified that the time series indeed behaves like 1/*f* noise (see “Methods”). In addition, we tested each of the ten simulations of the model from Fig. 6 (in purple) for the presence of 1/*f* noise, and we found that it is present in 80%. Also for the time series of the spine area from the longer experimental recordings (Fig. 4a), the method in Wagenmakers et al.^21^ confirms that the spine area evolution is dominated by 1/*f* noise (see “Methods”).

#### Self-organized criticality explains 1/*f* noise in spine size fluctuations of the model

To explain the presence of 1/*f* noise in spines, we explored which mechanism could be generating it in the model. One of the most common theories, proposed by Bak et al.^17^, is that 1/*f* noise emerges when a system is in a self-organized critical state where small fluctuations cause events of all sizes with a probability density function that is described by a power law. The duration of these events is related to their size; and hence, described by a power law function that correlates to 1/*f* noise. Therefore, this theory connects the temporal 1/*f* noise with the evolution of a spatial structure with scale-invariant, self-similar properties^22^.

In our model, the changes in spine size are due to the force exerted by polymerization of actin barbed ends at distinct foci. Hence, the fluctuations in spine area correlate to the number of barbed ends (significant Pearson’s correlation coefficient of 0.6751 with p-value $< 0.01$ using the data from Fig. 7c,e). Importantly, the branching probability depends on the membrane force and number of barbed ends in the foci. Therefore, it acts as a feedback mechanism that decreases the probability of branching if there are many barbed ends in the focus or if the membrane force raises. Figure 7e shows that the total number of barbed ends in our simulations behave like avalanches, in the sense that they have finite duration and different lengths and sizes. Hereinafter, we define the size of an avalanche in our system by the number of polymerization events. Since the barbed ends polymerize at each time-step, size is equivalent to the sum of barbed ends at each time-step during the avalanche (see Fig. 7f).

To examine whether our model has spatio-temporal power law characteristics, we collected all avalanches for ten 60-min simulations (corresponding to Fig. 6) and calculated the probability of their size and duration. Figure 7g,i show the log-log plot of such probabilities, which are for long stretch straight and have negative slope. Hence, the log-log probability plots behave like power law functions. The deviation from the straight line in Fig. 7i is due to the finite lifetime of the polymerization foci (finite-size effect^22^). Interestingly, as in Back et al.^17^, the mean of the 1/*f* noise exponent resulting from the ARFIMA fittings $\alpha \approx 0.72$ relates to the exponent of the power law of the event lifetime probability distribution $1.36 \pm 0.008 \approx 2-\alpha$, calculated as in Newman^23^. These features also arise in simulations with different sets of parameters (see Supplementary Information, Fig. S6). Moreover, various other tests confirm that our simulations show criticality (see Supplementary Information, Fig. S7). Therefore, we concluded that actin polymerization in the modeled dendritic spines self organizes to a critical state.

#### The critical state of the actin network in our model optimizes spine enlargement

To investigate whether actin polymerization operating in a critical state supports spine functionalities, we considered a version of our model, in which actin dynamics is non-critical, as control condition. The critical state is mainly determined by the feedback exerted by the barbed ends and the membrane force onto the branching rate. Therefore, for the control condition we “break” the feedback by considering a constant branching rate (see “Methods” for details) acquiring a non-critical model version as indicated in Fig. 7h,j. The log-log plots of size and lifetime probability distribution of actin avalanches without feedback are non-linear, failing to exhibit a power law relation.

**Figure 8:**
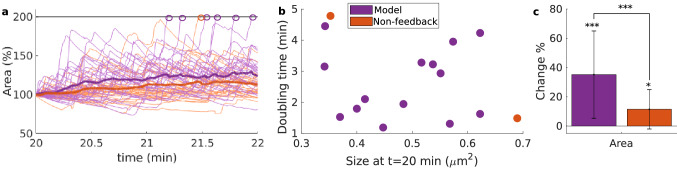
Spine size evolution upon LTP for different models. (**a**) Evolution of spine area changes. Circles mark the time point at which the spines double their area from that at minute 20, when there is an increase in the nucleation rate. The simulations corresponding to the model without feedback are color-coded in orange, whilst the simulations of the feedback-containing model are purple. (**b**) Time to doubling the spine area at minute 20. (**c**) Mean ± std of the percentage change of the area averaged over time. Time averages were taken 5 min before and after the change in nucleation rate. Asterisks: significance according to a Welsh’s test: *p-value = 0.05; ***p-value $<=\,0.01$.

Next, we examined the reaction of both models to an structural LTP-like event. It is known that during the first 5 min after LTP induction, F-actin rapidly dis- and re-assembles, leading to a substantial remodeling in the actin cytoskeleton that enlarges the spine^24^. As F-actin disassembly increases the quantity of G-actin that makes nucleation more likely (which triggers in turn the re-assembly of F-actin). Such an event was mimicked by increasing the nucleation rate. We performed 30 simulations of the model with and without the feedback mechanism in the branching rate. In these simulations the nucleation rate was increased by a factor of three at minute 20 such that each simulation constitutes a different spine. Figure 8a demonstrates that the spine area shows bigger and faster enlargement after an LTP-like event with feedback (purple) than without feedback (orange). Moreover, more spines in the model with feedback doubled their size within 5 min after this event. Note that in Fig. 8b there is a tendency of large spines to take longer to double their size. Importantly, within this time span, the spines of the feedback-model grow significantly larger than those without feedback (according to a Welsh’s test with p-value < 0.01, see Fig. 8c).

## Discussion

In the current study, we showed that our biophysical model of spine shape fluctuations replicates experimental data, and used it to make predictions on the actin dynamics in dendritic spines. To this end, we first developed measures to describe spine size and shape fluctuations and their statistics and matched model to data. Using our model, which implements the complex feedback between spine shape and the stochastic dynamics of actin polymerization, we investigated fast spine fluctuations on long-timescales, currently not well accessible in experiments. We find that size fluctuations follow 1/*f* noise and our model predicts that this might be due to a process of self-organized criticality of the actin dynamics, which allows the spine to react stronger and faster to changes in molecular dynamics, for example during LTP.

### Experimental data

In this study we analysed confocal microscopy data. Although super resolution images would have provided a more accurate quantification of spine morphology, the access to datasets with enough images and high enough temporal resolution is limited due to several problems. For example, in SIM, multiple images have to be acquired to reconstruct the final image which impairs time resolution. Similarly, in RESOLFT, the switching of the molecules requires pixel dwell times in the ms range. Therefore, the acquisition of an image with several spines is too slow (min range). On the other hand, STED nanoscopy has achieved imaging of individual spines with $\sim$ 10 s time resolution^5^. However, the signal-to-noise ratio is usually worse and the number of frames acquired with this technique is limited because it is prone to bleaching, which prevents tracking a spine for long times as required here. Considering all these practical limitations, confocal microscopy still appears as the best microscopy technique to address this question, even if finer fluctuations might be overlooked.

### New analysis methods to characterize spine shape and size

When studying fluctuations of a signal over time, the distinction between relevant information and noise is crucial. Here, we used time series analysis to detect whether there are certain characteristic tendencies in the dendritic spine size changes over time. Specifically, we used ARIMA models to analyse the experimental data. This novel approach appears, thus, promising for obtaining additional quantitative information from experiments by creating forecasts of the time series behavior over longer temporal periods^25,26^.

We observed that the shapes of dendritic spines are highly asymmetric, and hence, often employed, common shape factors; such as the circularity index or aspect ratio measures; fail to describe this well. Therefore, in order to better describe the evolution of spine shapes, we proposed new set of shape descriptors. Such descriptors are based on concepts of circular statistics^20^ that have been implemented, for example, to describe the asymmetry of visual cortical response curves to moving, oriented stimuli^27,28^.

We adapted these descriptors to characterize the directional *D* and orientational *O* selectivity of the spine shape. As indicated by Li et al.^27^, an important feature of these descriptors is their robustness to noise, because instead of using a perfect fit to the data to calculate them, only its most significant components are used. Moreover, these descriptors consider the entire shape of the spine head to calculate *D* and *O*, and are normalized with respect to the general size *S* to allow comparison with different spines. Therefore, *D* and 0 describe the overall shape characteristics.

We found that our shape descriptors fluctuate randomly over time in most of the cases. Moreover, using these new shape descriptors we showed that, in line with the previous findings of Fischer et al.^2^, shape changes are uncorrelated to size changes in dendritic spines. Importantly, this holds also for different data sets verifying the general character of this property.

### Spine size over long periods exhibit 1/*f* noise

We used our model to explore the behavior of spine size fluctuations for long durations and found the presence of 1/*f* noise. Moreover, we showed that 1/*f* noise is also present in longer experimental recordings. Interestingly, this type of noise has been previously identified in the brain at quite different temporal scales^29–32^. Furthermore, also in a human cognitive temporal estimation task, 1/*f* noise has been found^21^. Therefore, 1/*f* noise seems a ubiquitous feature of the nervous system.

### Actin in the spine self-organizes into a critical state

As a consequence, we explored which mechanism could generate 1/*f* noise in our modelled spines. Although the concept of self-organized criticality and its role in the brain has been controversial since its origin^33,34^, we chose this concept with the aim to link spatial and temporal phenomena in our model, as in the original interpretation^17^. Because blockage of actin polymerization in spines inhibits spine shape changes^2,3^, we proposed actin polymerization as the molecular mechanism that causes 1/*f* noise in our model. Hence, we studied the size and length of polymerization events in our model and found that they exhibit robust spatio-temporal power law correlations. Since this feature does not depend on the fine tuning of model parameters (see Supplementary Information, Fig. S6), we concluded that in the proposed model actin dynamics self-organize into a critical state. Similar results have been obtained by another study by Cardamone et al.^35^, who proposed and experimentally confirmed that growing actin networks in neural growth cones self-organized in a critical state.

The model predicts that there are polymerization *avalanches*, which occur in bursts originating from different foci. There is some indirect evidence that supports this. For example, Honkura et al.^7^ detected two distinctive pools of actin, a static and a dynamic one within the spine and Frost et al.^8^ observed that fast polymerization, corresponding to the dynamic pool, occurs at discrete foci. Thus, there is always a certain equilibrium existing between free actin monomers and actin filaments (F-actin and G-actin) in the spine. For example, Okamoto et al.^36^ observed that this equilibrium *globally* changes during spontaneous width changes of dendritic spines. We hypothesize that the existence of discrete polymerization foci^8^ shows that the equilibrium is also often *locally* disturbed by the gathering of monomers that bind and start a new filament. However, the amount of locally accessible free actin is finite, and hence, the lifetime of any focus and its filaments is limited (also due to capping processes, etc.). These experimentally confirmed processes can, thus, create the balance between new actin filaments outgrowth and their collapse required in our model to bring such a system into a self-organized critical state.

Similar local avalanche-like behaviour has also been found in a recent theoretical study^37^ that investigates the effects of the branching protein Arp2/3 on the contractility of the actomyosin network in dendritic spines. This contractility arises from the forces generated by the motors across actin filaments or from compression and expansion along the filaments due polymerization^37^, resulting in fluctuations in branched actin networks that produce local collapse events.

Here, we speculate that this creates a system, which is highly susceptible to change and can react efficiently, for example, when a spine will grow quickly following, for instance, the induction of long term potentiation^15,24,38^.

### Negative force-feedback

While these observations are phenomenological, the model suggests a possible biophysical mechanism that could underlie this. In general, a critical state is maintained by a constant flow of energy and material that is reached via feedback mechanisms^39^. Self-regulation in the model occurs through a feedback mechanism in the branching rate of the barbed ends, which depends on the local shape configuration and the number of barbed ends at each actin polymerization focus. The negative feedback between the branching rate and the force generated by the membrane is due to our assumption that the branching rate is proportional to the polymerization speed^14,40^, which slows down under stronger force^41^ (based on the Brownian ratchet theory^6,42^). Hence, this negative feedback in the branching rate is actually an indirect effect from the membrane force. Negative feedback mechanisms have been previously studied. For example, a study combining theory and experiments showed that an increase in membrane tension inhibits actin polymerization in neutrophil^43^. In amoeba^44^, neutrophil^45^ and HL-60^46^ motility models, a negative feedback between actin and nucleation-promoting factors give rise to traveling waves. Moreover, theoretical models show that different cell behaviours can emerge depending on the amount of actin negative feedback^47^.

However, recent experiments also showed a more direct regulation of branching by a counteracting force: Risca et al.^48^ showed that F-actin branching increases when the filaments bend in response to compressing forces of the membrane, producing a positive feedback between the branching rate and the membrane force. Our model could be extended to include filament bending and investigate whether it still exhibits self-organized criticality, but this would exceed the scope of the current study. Along this line, also the question whether the net effect of direct and indirect force-dependencies of branching gives rise to a positive or negative feedback for actin polymerization remains to be explored in further experimental and theoretical studies.

### Proposed experiments

In our model, global criticality emerges from the local properties of the different foci. This leads indeed to a balance between static filaments and free actin monomers. We show that in our model this balance is optimal to respond to an LTP-like event leading to a faster and stronger enlargement of the spine head compared to a spine with non-critical actin network. To validate this hypothesis, new experiments measuring the size and duration/lifetime of the actin polymerization foci for long timescales are needed. Such experiments are very challenging since the actin structure within a spine is dense, leading to signal saturation and difficulties in the visualization of single filaments even with the most advanced nanoscopy techniques. Furthermore, imaging at high spatial and temporal resolution for long timescales is impaired by the bleaching of the reporter molecules. Lastly, labeling strategies relying on the direct labeling of endogenous actin (e.g. SiR-Actin^49^) are based on Jasplakinolide and hence might interfere, even if only minimally, with the fine regulation of actin dynamics in the spines. Hence, an experiment to validate our hypothesis should aim at labeling only a sparse subset of actin, thereby enabling the visualization of small changes in concentration.

Besides fluorescence intensity, another possible readout is FRET, that could allow discriminating between F- and G-actin^36^. Sparse labeling could be achieved by short pulse-chase experiments in cells expressing actin in fusion with a self-labeling tag (e.g SNAP-Tag or HaloTag^50^) or by injecting a relatively low amount of labeled actin monomers. The optimal image acquisition rate for longer experiments can be estimated after studying the focus lifetimes over short periods of time but at high frequency. This could allow testing the self-regulation mechanism by tracking actin dynamics at a nucleation focus and measuring the extent of local spine deformation due to fast actin polymerization at a single focus and whether the focus eventually dies out.

## Methods

### Structure of the model

We are basing our model here on the 2D model with fixed PSD and neck described in Bonilla-Quintana et al.^14^. Note that in the older study a detailed verification against simulations in 3D (full spine volume mesh-model) is provided to show that the 2D reduced model captures the actin-membrane interaction accurately. We first describe the main characteristics of the basic model (for all details see Bonilla-Quintana et al.^14^) and then the extensions made for the current study.

The spine membrane is approximated by a mesh $\Gamma$ of *n* vertices at position ${\mathbf {x}}^k = (x^k,y^k) \in {\mathbb {R}}^2$, $k = 1,\ldots ,n$. At every time-step:

- A polymerization focus *i* nucleates at a random location ${\mathbf {X}}_n^i$ (close to the PSD) with probability $\gamma _f \Delta _t$.
- Each barbed end of F-actin polymerizes G-actin. Additionally,
  - Uncapped barbed ends branch creating a new filament with a probability $\Delta _t \gamma _{branch}(t)$.
  - Uncapped barbed ends cap with a probability $\Delta _t \gamma _{cap}$. Consequently, the barbed end stops generating force and, hence, the corresponding filaments are eliminated from the simulation.
  - Capped minus ends uncap with a probability $\Delta _t \gamma _{uncap}$.
  - Uncapped minus ends are eliminated with a probability $\Delta _t \gamma _{sever}$.
- Mesh vertices displace according to
  1 $$\begin{aligned} \frac{\mathrm {d}{\mathbf {x}}^k}{\mathrm {d} t} = \varsigma \left( {\mathbf {F}}_{mem}({\mathbf {x}}^k) + {\mathbf {F}}_{fil}(\mathbf{x} ^k)\right) . \end{aligned}$$

In Eq. (1) $\zeta$ is a proportionality constant, ${\mathbf {F}}_{fil}$ and ${\mathbf {F}}_{mem}$ are the actin and membrane forces, respectively. Here

2 $$\begin{aligned} {\mathbf {F}}_{fil}({\mathbf {x}}^k) = \sum _{i \in {1,2,\ldots n_f}} W(||{\mathbf {x}}^k-{\mathbf {X}}^i_c||)B^i(t) {\mathbf {V}}^{i,k}, \quad W(x) = \frac{\alpha }{\sigma \sqrt{2\pi }} \mathrm {e}^{-\frac{x^2}{2 \sigma ^2}}, \end{aligned}$$

where $\alpha$ denotes the amplitude and $\sigma$ the standard deviation of the Guassian kernel *W*, $n_f$ is the number of active actin foci with *B* barbed ends, and $\displaystyle {{\mathbf {V}}^{i, k} = \frac{{\mathbf {x}}^k - {\mathbf {X}}_n^i}{||{\mathbf {x}}^k - {\mathbf {X}}_n^i||}}$ the normalized direction vector of the force from focus *i*, located at ${\mathbf {X}}_n^i$.

The membrane force is given by

3 $$\begin{aligned} {\mathbf {F}}_{mem}({\mathbf {x}}^k) = - \frac{\partial {\mathcal {E}}_{mem}}{\partial {\mathbf {x}}^k} , \end{aligned}$$

where

4 $$\begin{aligned} {\mathcal {E}}_{mem} = P \Omega + \tau S + 2\kappa \int _\Gamma H^2 \mathrm {d}s \end{aligned}$$

is the Helfrich free energy. Here *P* is the difference between internal and external pressure, $\tau$ the line tension, and $\kappa$ the bending modulus. $\Omega$ denotes the area enclosed by the membrane, *S* the boundary length, and *H* the mean curvature.

All the rates are constant, except the branching rate

5 $$\begin{aligned} \quad {\gamma }^i_{branch}(t) = \phi k_{on}\delta a \exp \left( -\frac{|| {\mathbf {F}}_{mem}^i(t) || \delta }{ k_B T B^i(t)}\right) \frac{1}{B^i(t)}. \end{aligned}$$

that changes at every time-step and depends on the number of barbed ends at each focus *i*, $B^i$, and the membrane force. Here, *T* is the absolute temperature, $k_B$ the Boltzmann’s constant, $\delta$ the length of an actin monomer, $k_{on}$ the barbed end monomer assembly rate, $\phi$ the branching rate amplitude and *a* the concentration profilin-ATP-actin available for polymerization. As in Bennett et al.^40^, our branching rate is inversely proportional to the number of barbed ends. Hence, the number of barbed ends fluctuates around a fixed value because the branching rate increases when there are few filaments and decreases when the number of filaments increases. The branching rate is proportional to the treadmilling velocity, but instead of using a constant velocity, we employ that proposed by Mogilner and Edelstein-Keshet^41^ for lamellipodia in moving cells : $\displaystyle {V = k_{on}\delta a \exp \left( -\frac{f \delta }{ k_B T}\right) }$, where *f* denotes the force per barbed end. Unlike in lamelipodia, the shape of spines cannot be approximated by a straight line. Therefore, we propose $f= || {\mathbf {F}}_{mem}^i(t) ||/B^i(t)$ to account for the local geometry and number of barbed ends within a polymerization focus. Due to these assumptions on actin and Arp2/3 dynamics that are based on previous models, our branching rate depends on the number of barbed ends and the force generated by the spine membrane in a complex manner, which is depicted in Supplementary Information Fig. S1. All parameters are the same as in Bonilla-Quintana et al.^14^, except for $\sigma = 0.2$ (see Supplementary Information, Table S3).

This basic model has been extended here to incorporate the fact that the PSD and the neck can change (on a slow timescale), too. Thus, the PSD lateral movements and size changes used in the current study are implemented by introducing four random processes:

- The PSD right boundary elongates (moves to the right) with probability $\Delta _t \gamma _{r+}$.
- The PSD right boundary shrinks (moves to the left) with probability $\Delta _t \gamma _{r-}$.
- The PSD left boundary shrinks (moves to the right) with probability $\Delta _t \gamma _{l-}$.
- The PSD left boundary elongates (moves to the left) with probability $\Delta _t \gamma _{l+}$.

For this, we draw a number from a uniform distribution for each process. If the number is less than the designated probability, then the PSD elongates and/or shrinks, respectively. Such events are implemented by adjusting the vertices in the spine mesh corresponding to the PSD. When the PSD right (left) boundary shrinks, the first (last) vertex corresponding to the PSD is released, in the sense that no longer forms part of the PSD and can move according to the forces generated by actin and the membrane. When the PSD right (left) boundary elongates, the PSD incorporates the neighboring vertex to the right (left) from the spine. Consequently, the vertex is fixed with the *y*-coordinate set to $h_{PSD}$. Note that these events are independent, and thus, can result on a lateral displacement of the PSD with respect to the neck.

Likewise, the PSD moves vertically upward by setting $h_{PSD} = h_{PSD} + \Delta d_{mov}$ with probability $\Delta _t \gamma _{up}$, and moves vertically downward decreasing $h_{PSD}$ by $\Delta d_{mov}$ with probability $\Delta _t \gamma _{down}$. If the PSD moves vertically, all the corresponding vertices in the mesh change their *y*-coordinate to the new value of $h_{PSD}$.

Since the neck center is the reference point to calculate the here-proposed shape descriptors, we prefer to avoid its displacement by only allowing symmetrical neck width changes. For this, we elongate the neck with probability $\Delta _t \gamma _{n+}$ by recruiting the right and left neighboring spine vertices and fix them to $h_{neck}$, or shrink the neck with probability $\Delta _t \gamma _{n-}$ by releasing the initial and final vertices corresponding to the neck.

Note that all these events are independent. We select $\gamma _{\blacklozenge } = 1/75$ $\text {s}^{-1}$, $\blacklozenge \in \lbrace r+,r-,l+,l-,n+,n-, up, down \rbrace$, which renders a good qualitative fit to the data. For $d_{mov}$, we use the same value as the minimum distance allowed between mesh vertices $d_{min} = 0.018$ $\mu$m. In the simulation, the changes to the PSD and neck are computed after the actin dynamics.

#### Non-feedback model

For the non-feedback model, we modify the branching rate in Eq. (5) to have a constant membrane force $|| {\mathbf {F}}_{mem}^i(t) || = || {\mathbf {F}}_{mem} || = 7 \mathrm {pN}$ and number of barbed ends $B^i(t) = B = 4$. These quantities correspond to the membrane force at the most likely nucleation locations in the initial (resting) shape and the mean number of barbed ends of 100 Monte Carlo simulations with fix ${\mathbf {F}}_{mem}$ and random initial $B \in [1,20]$.

### Experimental data analysis

We analyze experimental data provided by Dr. Mikhaylova that corresponds to Figs. 6 and S7 in^16^ and consists of time-lapse confocal microscopy images taken every 10 s for 5 min from wild type mouse hippocampal primary cultures at 12 days in vitro (DIV12) co-transfected with mRuby2 (volume marker) and GFP-actin.

We also analysed a different data set from the lab of E.D., which consist of 100 frames taken every 9.6 s. For this, hippocampal neurons cultured on poly-ornithine-coated coverslips were stained at 19 DIV with 2 $\mathrm {\mu g/ mL}$ DiO (Thermo Fisher) for 20 min in ACSF, washed for 10 min and imaged in confocal mode in ACSF buffer without any potentiating compound at room temperature.

The datasets used for our analysis have been acquired with high magnification and high numerical aperture objective lenses. Therefore, the anticipated resolution is around 200 nm. In particular, the experimental setup in which the imaging of our second dataset was performed, the anticipated resolution is approximately 180 nm (488 nm excitation, objective lens with 1.4NA).

We process these images using Fiji (Version 2.0.0^19^, http://fiji.sc, RRID: SCR_002285). First, we trace a square region of interest (ROI) around a spine and duplicate the entire stack of images in the GFP-actin channel. We apply a Kuwahara Filter with a kernel size of 3, which preserves edges; and, thus, the small protrusions of the spines. The resulting image is thresholded (using the default method) and then a mask is created in which a watersheed segmentation is applied to separate the spine head from the neck. Finally, a ROI is created around the spine using the Analyze Particles function and the neck position is manually traced (see Fig. 2a, and Supplementary Information Fig. S2 for further details on image analysis). Subsequently, we load the ROIs into Matlab (Version R2017b, MathWorks, http://www.mathworks.com/products/matlab/, RRID:SCR_001622) using the function ReadImageJROI.m^51^.

#### Shape descriptors to quantify shape fluctuations

To calculate the shape descriptors, we use the spine neck center as the origin point and trace a ray every 15$^{\circ }$ ($\approx 0.2618$ rad). If the ray corresponding to the angle $\theta _i$ intersects the ROI, then a sample point is taken at the intersection (black dot in Fig. 3a) and the distance to neck center is measured ($\text {dROI}(\theta _i)$ brown line in Fig. 3b). If the traced ray does not intersect the ROI, then $\text {dROI}(\theta _i) = 0$. Thus, $\text {dROI}(\theta )$ accounts for the distance from the neck center to the spine membrane at the sample points. Note that $\text {dROI}(\theta )$ has a $2 \pi$ period and each spine has the same number of sampled points regardless of size. Following Li et al.^27^, to obtain an approximation $R(\theta )$ for $\text {dROI}(\theta )$ (orange dotted line in Fig. 3b), we perform an optimal periodic regression on the distance between the neck and sample points of the spine $\text {dROI}(\theta )$ (brown line in Fig. 3b). First, an “entire fitting” of $\text {dROI}(\theta )$ is computed by

6 $$\begin{aligned} \text {dROI}(\theta ) = A_0 + \sum _{k=1}^{n-1} \left( A_k \cos (k \theta ) + B_k \sin (k \theta )\right) + A_n \cos (n \theta ), \end{aligned}$$

where the regression coefficients ($A_k$, $B_k$) are given by

7 $$\begin{aligned} A_0 = \frac{1}{2n} \sum _{i=1}^{2n} \text {dROI}(\theta _i) , \quad A_n = \frac{1}{2n} \sum _{i=1}^{2n} \text {dROI}(\theta _i) \cos (n \theta _i),\nonumber \\ A_k = \frac{1}{n} \sum _{i=1}^{2n} \text {dROI}( \theta _i) \cos (k \theta _i), \quad B_k = \frac{1}{n} \sum _{i=1}^{2n} \text {dROI}(\theta _i) \sin (k \theta _i). \end{aligned}$$

Here, $i=1,2, \ldots , 2n$ is the number of sampling points corresponding to the angle $\theta _i = (i-1) \pi / n$, with $n=12$. As in Li et al.^27^, we assume that an entire fitting to the data is improper due to the errors that can arise from the experiment or the data processing. Therefore, all the terms with a standard regression coefficient smaller than 0.1 are discarded (one by one) if they do not significantly alter $\text {dROI}(\theta )$ (assessed using a F-test with a level of significance of 0.1). The resulting function $R(\theta )$ is periodic and, hence, can be divided in three parts:

8 $$\begin{aligned} R(\theta ) = S + D(\theta ) + O(\theta ), \end{aligned}$$

where

9 $$\begin{aligned} S= & {} A_0, \nonumber \\ D(\theta )= & {} \sum _k (A_k \cos (k \theta ) + B_k \sin (k \theta )), \quad k=1,3, \ldots ,k \le n, \nonumber \\ O(\theta )= & {} \sum _k (A_k \cos (k \theta ) + B_k \sin (k \theta )), \quad k=2,4, \ldots ,k \le n, B_n = 0, \end{aligned}$$

and the discarded terms are set to zero (Fig. 3c). Figure 3b shows the resulting function $R(\theta )$. These parts correspond to general size *S* of the spine (constant offset in the Fourier series), the preference of the spine to tilt along a certain direction $D(\theta )$ (Fourier components with a odd number of peaks, Fig. 3c orange) and the orientational selectivity of the spine head $O(\theta )$ (Fourier components with an even number of peaks, Fig. 3c green). In order to describe the overall spine shape, the contributions of $D(\theta )$ and $O(\theta )$ have to be considered over the full period. Moreover, to compare different spines with different sizes, $D(\theta )$ and $O(\theta )$ should be calculated in terms of *S*. Thus, following Li et al.^27^, these quantities are integrated and averaged:

10 $$\begin{aligned} D = \frac{1}{2 \pi } \int _0^{2 \pi } \left| D(\theta )\right| \mathrm {d}\theta \times \frac{100}{S}, \quad O = \frac{1}{\pi } \int _0^{\pi } \left| \frac{O(\theta )}{\mathrm {d}\theta }\right| \mathrm {d}\theta \times \frac{100}{S}. \end{aligned}$$

Note that *D* and *O* are expressed in terms of percentage of *S* and percentage of *S* per radian, respectively. These descriptors can thus characterize asymmetrical shape configurations. For example, *D* is bigger if the spine head considerably tilts towards one direction (from the spine neck), whilst *O* has larger values if the spine elongates along one or more directions. For further interpretation of these descriptors, see Supplementary Information (Fig. S1).

### Quantification and statistical analysis

The tendency analysis on the spine area and shape descriptors is performed in RStudio (Version 3.4.4^52^, http://www.rstudio.com/, RRID: SCR_000432) using the auto.arima() function with max.p=1, max.q=1, max.d=1, start.p=0, start.q=0, to ensure the selection of simple models, and assessed by the Akaike information criterion (AIC). We test if the residuals of the selected model are significantly stationary according to the augmented Dickey-Fuller test (adf.test() with 0 lag and p-value $< 0.01$). Moreover, the residuals’ autocorrelation (ACF) and partial autocorrelation (PACF) function are plotted. If the residuals of the selected model are non-stationary and/or have pronounced peaks in the ACF or/and PACF, we investigate which of the possible 8 ARIMA models best fit the data using the arima() function and AIC.

To test the presence of 1/*f* noise in the time series corresponding to the spine area, we use the method developed by Wagenmakers et al.^21^. This method is based on the idea that 1/*f* noise (related to long term correlations) appears between white noise (ARIMA(0,0,0)) and a random walk (ARIMA(0,1,0)).

Note that white noise, denoted by $X_t = \epsilon _t$, where $X_t$ is the value of an observation at time *t* and $\epsilon _t$ denotes a random number drawn from a normal distribution, is obtained taking the difference between successive observations in a random walk, given by $X_t = X_{t-1} + \epsilon _t$. This is capture by the parameter *d*, which denotes the number of difference needed for stationarity, in their corresponding ARIMA(*p*, *d*, *q*) model. Moreover, the spectral density of a random walk has a power law exponent of $\alpha = 2$, and the white noise has $\alpha = 0$, thus $d = \alpha /2$. This implies that, to model 1/*f* noise, $d>0$ must include fractional numbers, as in ARFIMA (autoregressive fractionally integrated average) models.

Following Wagenmakers et al.^21^, we test whether the spine area time series presents long-range or short-range dependencies, i.e., whether the time series fits better an ARFIMA(1,*d*,1) or an ARIMA(1,0,1) model. We apply the fracdiff() function in RStudio with nar=1, nma=1, to get the coefficients of the ARFIMA(1, *d*,1) model, and check if they are significantly greater than zero using summary() with signif.stars = TRUE. A time series exhibits 1/*f* fluctuations if *d* is significantly different from zero and if the AIC of the ARFIMA(1,*d*,1) model is greater than that of an ARIMA(1,0,1) model. For calculating the AIC of the ARIMA(1,0,1) model, we use the arima function. The residuals of both models are tested for stationarity as above. Particularly, for the area trace in Fig. 5b the AIC of the ARIMA(1,0,1) and ARFIMA(1,0.3145,1) fittings are -959.64 and -961.9958, respectively. For the longer experimental area trace in Fig. 4a the AIC of ARIMA(1,0,1) is -236.73 and for ARFIMA(1,0.3091,1) is -267.4099.

## Supplementary Information

Supplementary Information.

## Supplementary Information

The online version contains supplementary material available at 10.1038/s41598-021-83331-9. The code for the simulations is available from Github (https://github.com/MayteBQ/Critical-Actin-in-Dendritic-Spines).
