# Supplementary material for: Reproducing asymmetrical spine shape fluctuations in a model of actin dynamics predicts self-organized criticality
Source: Sci Rep. 2021 Feb 17;11:4012. doi: 10.1038/s41598-021-83331-9 (PMC7889935; doi:10.1038/s41598-021-83331-9)
Supplement: Supplementary file 1 — Supplementary Information. [file 41598_2021_83331_MOESM1_ESM.pdf]

Reproducing Asymmetrical Spine Shape Fluctuations in a Model of Actin Dynamics Predicts Self-organized Criticality

Supplementary Information

Mayte Bonilla-Quintana[1,*], Florentin Wörgötter[1,2], Elisa D'Este[3], Christian Tetzlaff[1,2] and Michael Fauth[1]

[1]University of Göttingen, Department for Computational Neuroscience, Göttingen, 37077, Germany

[2]University of Göttingen, Bernstein Center for Computational Neuroscience , Göttingen, 37077, Germany

[3]Max-Planck-Institute for Medical Research, Optical Microscopy Facility, Heidelberg, 69120, Germany

[*]corresponding author: mayte.bonilla-quintana@phys.uni-goettingen.de

Barbed ends branching rate

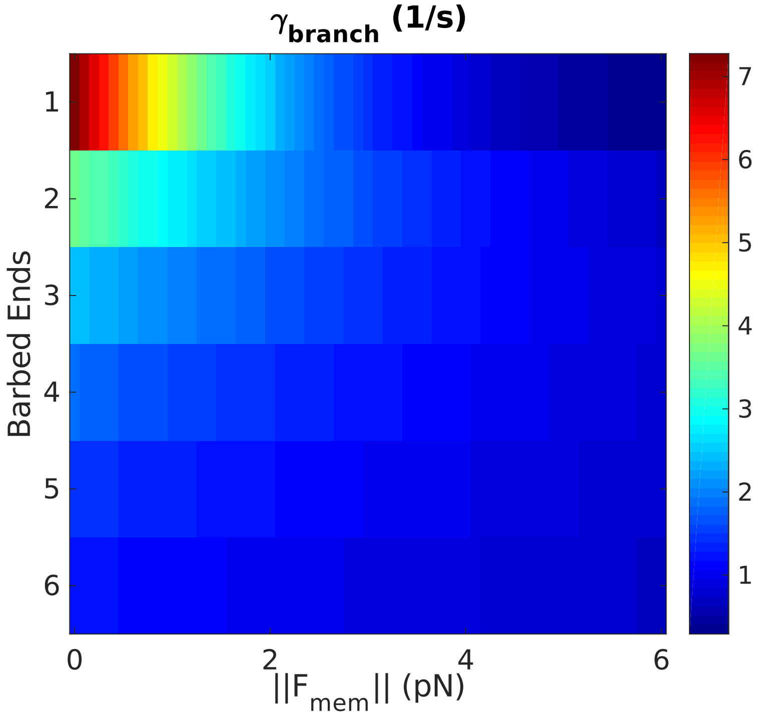

Figure S1: **Rate of branching of the actin barbed ends γ_{branch},** as in Eq. 5, varying the number of barbed ends and the force generated by the membrane $\mathbf{F}_{mem}$. The branching rate increases for small number of barbed ends and small value of the membrane force and decreases as these values increase.

Image analysis

Here, we describe in detail the steps of the data analysis. For this, we use the image of Figure 2 at t=50 s. First, we trace a rectangular ROI around the spine that encloses it at any time point (Fig. S2a). For every frame (Fig. S2b), we apply a Kuwahara filter, a noise-reduction filter that preserves edges. Figure S2c shows the resulting smoother image in which noise raising from the heterogeneous distribution of fluorophores inside the spine, which produces a mix of dark and bright areas, has been reduced. Also, the region corresponding to the spine is more homogeneous and distinct to the background while conserving the edges. To separate the foreground from the background, we threshold the image and create a mask (Fig. S2d) in which we run the watershed method that automatically finds the center of the spine head and separates it from the dendrite (Fig. S2e). To draw the ROI around the spine head, we use Analyze Particles (Fig. S2f). Finally, we overlay this ROI in the original image to manually identify the neck (Fig. S2g).

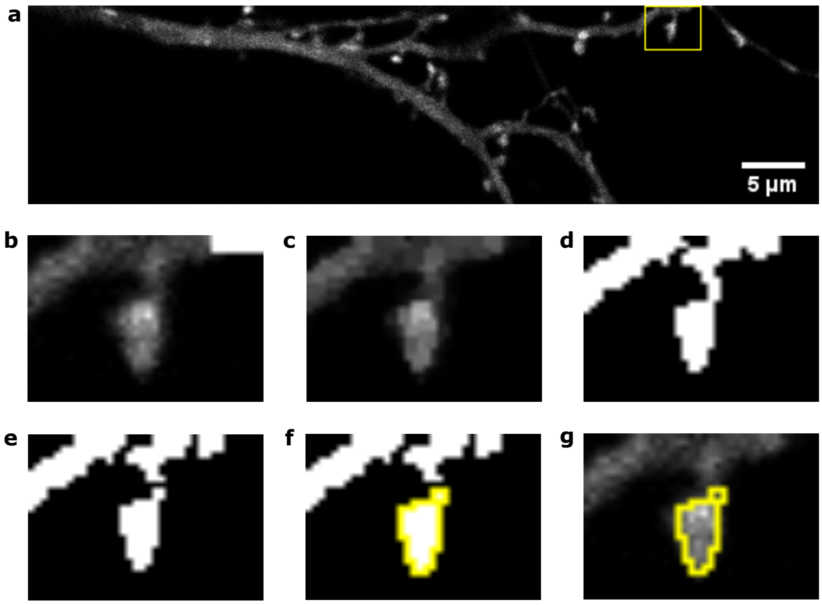

Figure S2: **Data Analysis Protocol**. **a**, original image, **b**, image corresponding to the square ROI in panel (a). Here, the bar on top corresponds to 1 μm. **c**, image after applying the Kuwahara filter and **d** after thresholding. **e**, final image after watershed segmentation and **f** same image with the resulting ROI. **g**, overlay of the original image from (b) with the ROI in (f).

ARIMA fittings

Table S1: **ARIMA FITTINGS**

Model	Area	S	D	O
Spine 1	WN	WN	WN	WN
Spine 2	DA	ES	AR	ES
Spine 3	DA	DA	WN	WN
Spine 4	MA	MA	AR	AR
Spine 5	AR	AR	MA	WN
Spine 6	RW	RW	WN	ES
Spine 7	ES	RW	WN	WN
Spine 8	ES	ES	MA	AR
Spine 9	ES	WN	WN	WN
Spine 10	AR	WN	MA	WN
Spine 11	MA	ES	WN	ES
Spine 12	AR	AR	ES	ES
Spine 13	WN	WN	WN	MA
Spine 14	DA	DA	WN	MA
Spine 15	AR	RW	ES	WN
Spine 16	AR	AR	RW	WN

Abbreviations:
WT: ARIMA(0,0,0), fluctuations in the time series are white noise around a mean value.
AR: ARIMA(1,0,0), the time series tends to return to a mean value.
MA: ARIMA(0,0,1), time series value at time t depends on the current and previous noise.
RW: ARIMA(0,1,0), the time series behaves like a random walk.
ES: ARIMA(0,1,1), the time series tends to fluctuate around a slow moving average.
DA: ARIMA(1,1,0), fluctuations in the time series depend on the past values as well as the difference between the past and previous values.

Shape Descriptors

In the following shape descriptors are interpreted. To achieve this, five different kinds of spine heads are depicted (left column of Fig. S3) and their shape descriptors are plotted (right column of Fig. S3).

S corresponds to spine head size

Note that S corresponds to the first term of $R(\theta)$ in Eq. (8) (see Fig. S3, center column, dashed line). Since $R(\theta)$ is periodic, S represents the average value of the function over all the domain ($\theta \in [0, 2\pi)$). Hence, S gives an average of the distance from the spine neck center to the membrane of the spine head at the sampling points. In this way, S can be used as an indicative of the spine head area. Moreover, there is a correspondence between area and S values in the sample spines: bigger spines have larger S value, and vice versa.

D indicates direction selectivity

Note that $D(\theta)$ in Eq. (8) is an odd function (see Fig. S3, center column, dotted-dashed line). For a "normal" spine shape (Fig. S3a), the maximum distance from the spine neck is assumed to be aligned with the neck center. Thus, the maximal value of $D(\theta)$ is $\approx \theta = \pi/2$. Moreover, the shape of $D(\theta)$ is similar to a sinusoidal function with period 2π and amplitude $\approx \pi S/2$. Therefore, when $D(\theta)$ is integrated and averaged (see Eq. (10)) it gives $D \approx 100\%$ of S.

Importantly, when the spine head shows a clear preference for a direction from the spine neck, $D(\theta)$ has a clear peak that resembles a sinusoid and, thus, $D \approx 100\%$ of S (see Figs. S3a-c). If all the sampling points are equidistant to the spine neck (Fig. S3d) then the amplitude of $D(\theta)$ is close to S. Hence, $D < 100\%$ of S. Likewise, $D < 100\%$ of S if the spine head has various bumps, which have similar distance from the neck (Fig. S3e). In this case, $D(\theta)$ peaks at the bump locations; however, these peaks are around S and the integration results in a smaller percentage of S. Hence, D is an indicator for spine head preference of direction from the spine neck. If the spine is centered or leans towards a side, then

$D \approx 100\%$. If the spine head membrane is equidistant to the neck or if the spine head has several bumps, then $D < 100\%$.

O indicates orientation selectivity

$O(\theta)$ is an even function (see Fig. S3, center column, solid line). Accordingly, only half of the period is considered when calculating O (see Eq.(10)). Note that O is the averaged integral over the derivative of $O(\theta)$ (see Eq. (10)). Therefore, it represents the changes in the slope of $O(\theta)$ between sampling points. For example, for a “normal" centred spine head shape (Fig. S3a), $O(\theta)$ is close to a cosine function with period 2π and amplitude $2S$. Thus, the changes of its slope between sample points have to be of the same magnitude and $O \sim 4S/\pi$.

When the spine head is more elongated with some orientation (Figs. S3b-c), then the slope of $O(\theta)$ is steeper and $O > 400/\pi$. For sharper elongations, O is larger (Fig. S3b). Likewise, when there are several sharp protrusions (Fig. S3e) the value of O is larger. Finally, if the spine head does not have any elongation (Fig. S3d), then $O(\theta)$ is almost constant and consequently, O is small. Therefore, O denotes spine elongations that are not reflected in known measures like the circularity index.

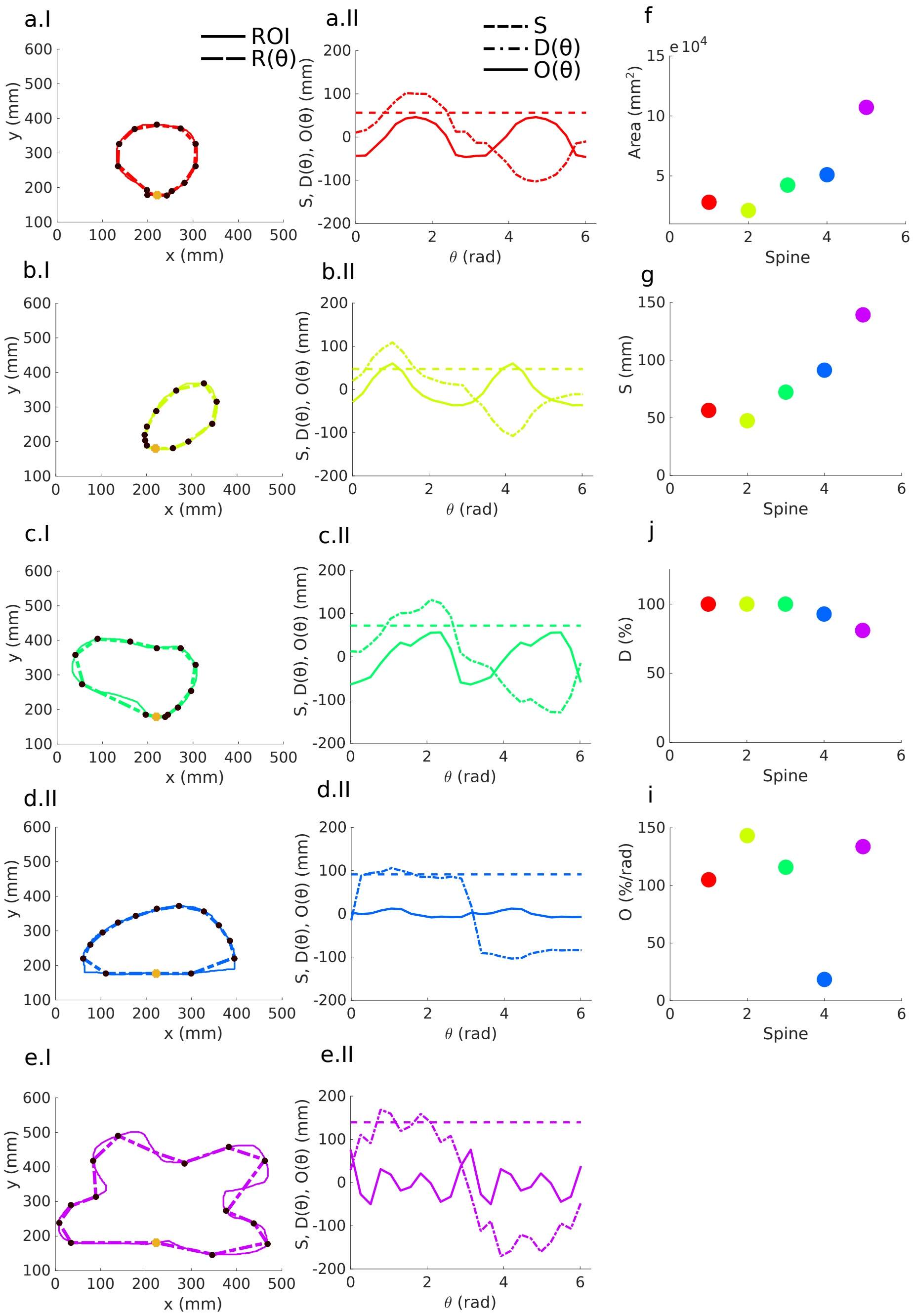

Figure S3: **Shape descriptors**. Left column: Different spine head shapes (solid line) and their approximation function $R(\theta)$ from Eq. (8) (dashed line). Black asteriks represent sample points and yellow dot the neck center. Center Column: Different parts of $R(\theta)$. Here the dashed, dotted dashed, and solid lines represent S, $D(\theta)$, and $O(\theta)$, respectively. Right column: Value of shape descriptors for the different spines (color-coded).

Difference between model and experimental data

Experimental data suffers from some inaccuracies due to the imaging method used. For example, in confocal microscopy images reflect the florescence of certain particles that are not equally distributed and appear blurred, depending on the quality of the microscope. To replicate such effects in our modeled spines, we randomly allocate fluorophores inside the spine shapes, represented by small circles (see Fig. S4a) colored with a dark grey. The inside of the spine and the figure background are colored in different shades of grey to mimic experimental recordings. Then, the image is blurred using the `imgaussfilt` function in MATLAB with $\sigma = 1.99$, which corresponds to the standard deviation of the point spread function calculated from the spines corresponding to the top stack in Figure 2b using Fiji. The resulting images are imported to Fiji and a ROI is traced, as in the experimental data. Next, the shape descriptors are calculated in MATLAB (see values in Table S2). Note that the values from the blurred images change considerably from those of the model.

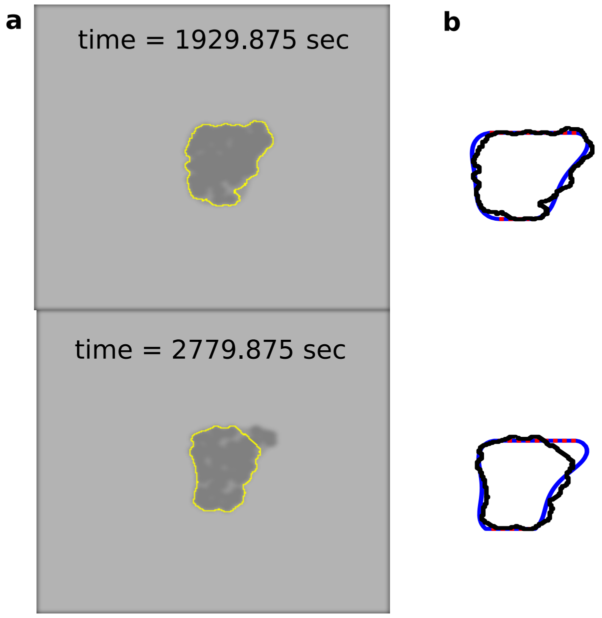

Figure S4: **Model replicating imaging effects**. **a**, model spine shape from the simulation in Figure 5 with fluorophores (circles) after filtering. Yellow line corresponds to the traced ROI following the methology described in Methods. **b**, extracted ROI from (a) and original shape. Red dotted lines correspond to the neck and PSD.

When analyzing all the blurred sample shapes of the simulation, we observe an increase in the coefficient of variance. However, the ROIs extracted from experimental data using the mRuby2 channel and the merged image show similar CVs, thus, the variance is conserved independently of the position of the fluorophores' locations (see Fig. S5). Therefore, we assume that the variability difference between experimental data and simulations is due to inaccuracies in the imaging method.

Table S2: **Shape Descriptors**

**Spine**	**Area** (μm^2)	**S** (μm)	**D (%)**	**O (%/rad)**
Spine 1	0.4806	0.2434	96.6792	96.6372
Spine 1 Blurred	0.4701	0.2535	84.0461	98.0735
Spine 2	0.4353	0.2325	94.7640	111.9447
Spine 2 Blurred	0.4070	0.2367	87.9546	97.2049

Spine 1 corresponds to Figure S4a at 1929.875 seconds.
Spine 2 corresponds to Figure S4a at 2779.875 seconds.

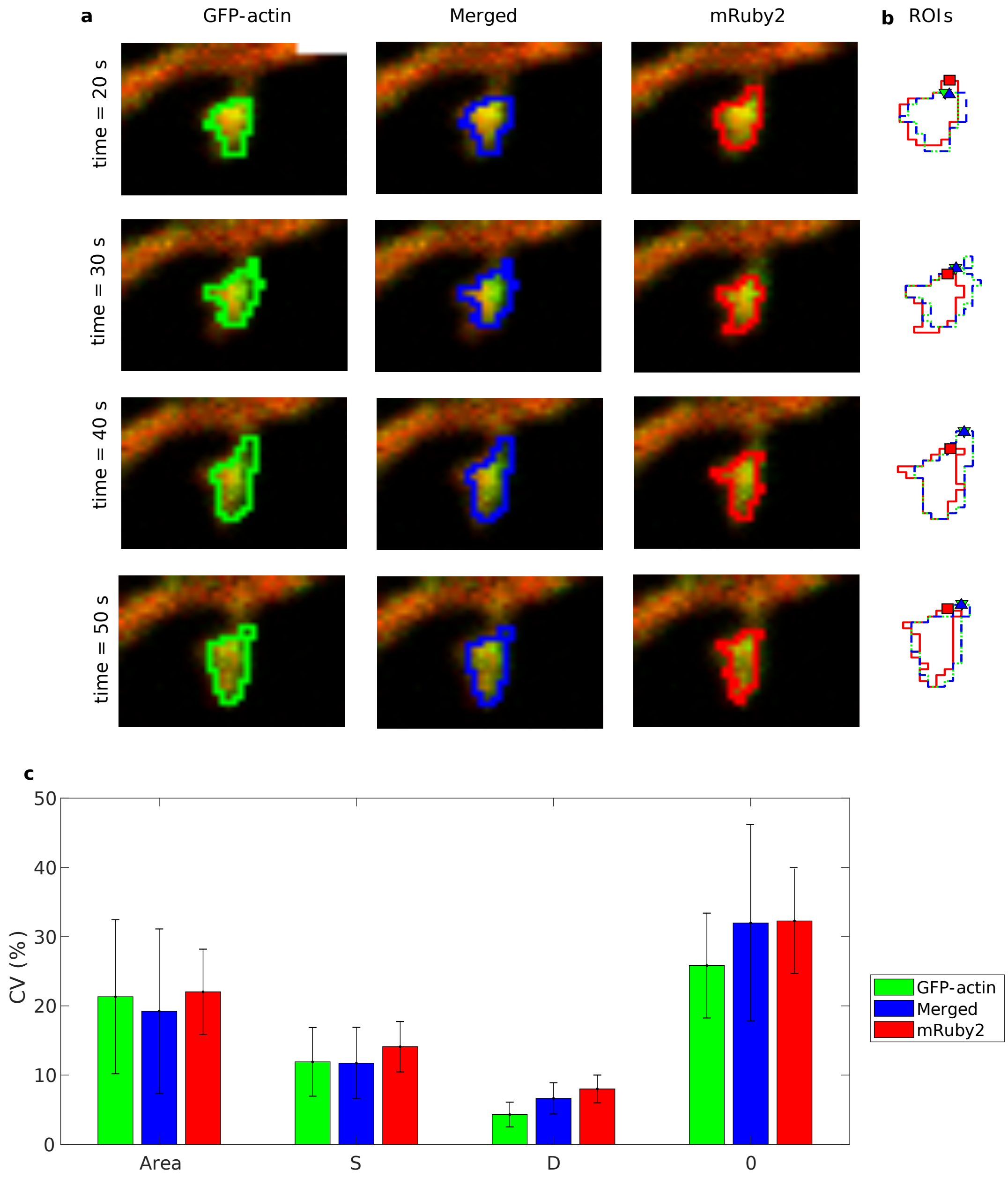

Figure S5: **ROIs from experimental data**. **a**, ROIs extracted from experimental data usign the GFP-actin channel (right), as in Figure 2, mRuby2 channel (left) and both channels merged (center). **b**, ROIs from (a) overlapped. **c**, median ± standard deviation of the coefficient of variability for shape descriptors calculated for each spine in the experimental data.

Self-organized Criticality Validation

We test whether the model self-organizes to a critical state without parameter fine-tuning. For this, we vary the branching rate amplitude ϕ that linearly relates to the branching probability, given by Eq. (5) (see Methods). Hence, ϕ modifies the number of barbed ends. Figure S6 shows that the log-log plots of the probability of avalanche size and lifetime show a power law behaviour.

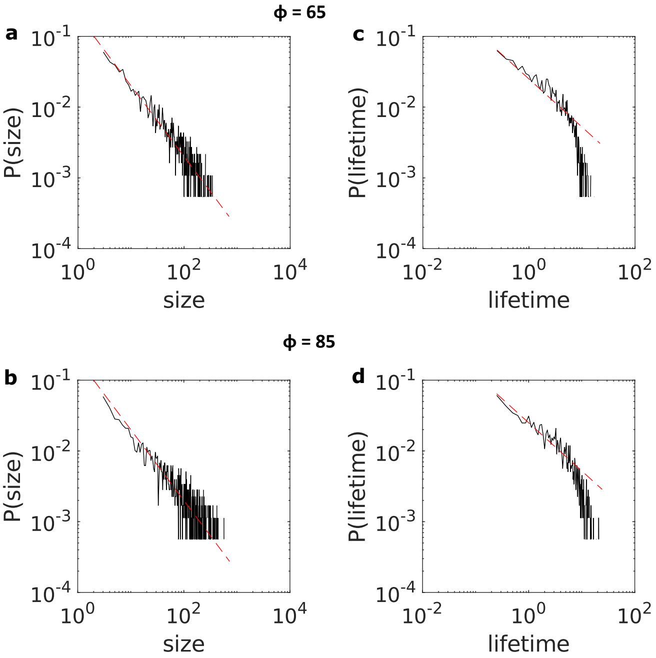

Figure S6: **Self-organized criticality under parameter variation**. Top $\phi = 65$, bottom $\phi = 85$. Log-log plot of the probability of avalanche size **a-b** and lifetime **c-d** corresponding to the data from 10 simulations of the model with the corresponding parameters. Red dotted lines correspond to straight lines with slope of -1 (a-b) and -0.68 (c-d).

We performed additional criticality tests to the simulations, as in [1]. First, we test if the system is temporally scale-free by changing the size of the time bins. Figure S7a-c shows that the avalanche size in simulations with different parameters had power law distribution regardless of the time bin. To test whether the system is spatially scale-free, we assume that, due to experimental limitations, we observe only two or one foci at a time. Thus, the avalanche size is calculated only with those foci: if a third focus starts when two are active, the third one is discarded and the barbed ends of that focus are not included in the avalanche size

calculation. The avalanche size distribution in Figure S7d-f exhibits a power law relation, hence, the system is spatially scale-free.

To evaluate if the interval between avalanches displays a scale-free behavior, two variables are introduced: s_c the critical size of an avalanche and η_c the waiting time between to avalanches with size bigger than s_c. To compare the distributions of η_c given s_c, we need to re-scale η_c, because larger avalanches are less probable. Thus, $\eta_c \to \eta_c R(s_c)$, with $R(s_c)$ the rate of having an avalanche bigger than s_c per time unit, and $P(\eta_c, s_c) \to P(\eta_c, s_c)/R(s_c)$. Note that after re-scaling the distributions in Figure S7g-i collapse into a single function F, hence $P(\eta_c, s_c) = R(s_c)F(\eta_c s_c)$. Note that F is a power law, confirming that the intervals between avalanches are scale-free.

Finally, we obtain the Allen Factor to assess criticality because it allows calculation of power law exponents α in the range of $0 < \alpha < 3$ [2]. We define $S_z(T)$ as the number of polymerizing events contained in a time window z of length T and the Allen Factor

$$AF(T) = \frac{\langle (S_{z+1}(T) - S_z(T))^2 \rangle}{2\langle S_z(T) \rangle}, \tag{1}$$

where $\langle \cdot \rangle$ denotes the expectation value. In short, the Allen Factor measures the degree of clustering of the polymerization events compared to a homogeneous Poisson point process, in which $AF(T) = 1$, $\forall T$. Note that in Figure S7j-k the Allen Factor has power law behavior for several time windows T, which indicates a scale-free point process.

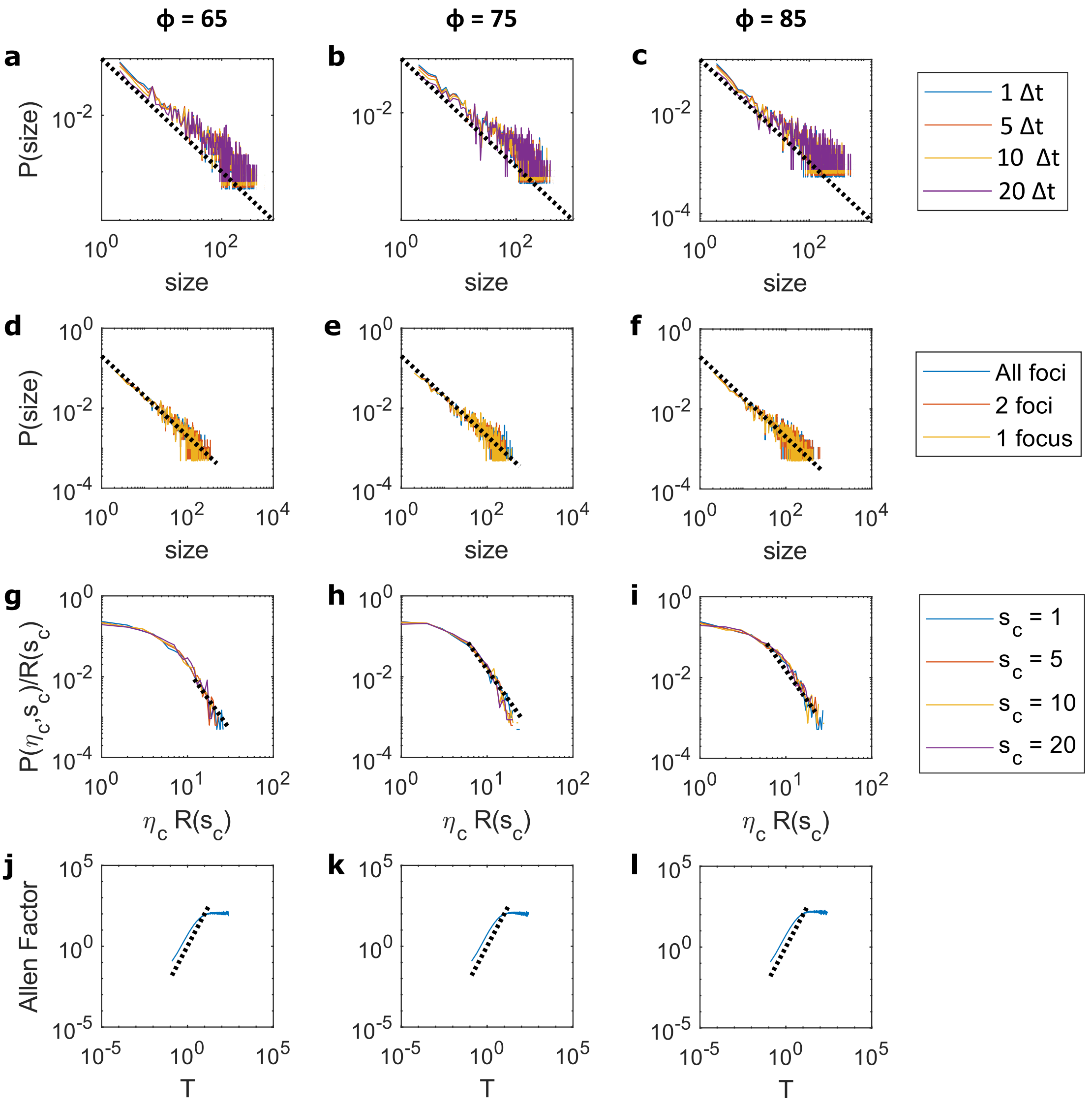

Figure S7: **Self-organized criticality tests**. Left, center, and right correspond to simulations with $\phi = 65$, $\phi = 75$, and $\phi = 85$, respectively. Log-log plot of the probability of avalanche size taking different time bins **a-c**, and restricting the number of foci **d-f** for the measurement. **g-i**, probability of interval duration η_c between avalanches of size bigger than s_c (see text for details). **j-l**, Allen Factor for different time windows T. Black dotted lines correspond to straight lines with slope of -1 (a-f), -3 (g-i), and 2 (j-l). Data from 10 simulations of the model.

Model Parameters

Table S3: **Model Parameter Values.**

Symbol	Unit	Definition	Value
Δ_t	s	length of the time-step	1/8
δ_s	μm	target length of and edge	0.03
r_{neck_0}	μm	initial neck radius	0.0995
r_{PSD_0}	μm	initial PSD radius	0.3571
h_{neck_0}	μm	initial value for fixing the neck	-0.49
h_{PSD_0}	μm	initial value for fixing the PSD	0.35
n_{f_0}	1	initial number of nucleation points	4
γ_{cap}	s^{-1}	barbed-end capping rate	1
γ_{uncap}	s^{-1}	uncapping rate for - ends	1/30
γ_{sever}	s^{-1}	depolimerisation/Severing rate of - ends	1
γ_f	s^{-1}	nucleation rate of new actin focus of activity	0.1
a	μM	concentration of profilin-ATP-actin at steady state	3.8
ϕ	μm^{-2}	proportionality constant	75
k_{on}	$\mu M^{-1} s^{-1}$	barbed-end monomer assembly rate constant	11.6
δ	μm	length of an actin monomer	0.0022
$k_B T$	pNμm	thermal energy	0.0041
P	pNμm^{-2}	difference between internal and external pressure	85.7143
τ	pNμm^{-1}	surface tension	15
κ	pNμm	bending modulus	0.18
α	pN	strength of filament influence	3.8
σ	1	extend of filament influence	0.2
ζ	$\mu m^2 s^{-1} pN^{-1}$	strength of force update	0.002
λ	μm	nucleation distance parameter	0.025

Table Modified from [3].

References

[1] Tetzlaff C, Okujeni S, Egert U, Wörgötter F, Butz M. Self-organized criticality in developing neuronal networks. PLoS Computational Biology. 2010;6(12).

[2] García-Marín A, Jiménez-Hornero F, Ayuso J. Applying multifractality and the self-organized criticality theory to describe the temporal rainfall regimes in Andalusia (southern Spain). Hydrological Processes: An International Journal. 2008;22(2):295–308.

[3] Bonilla-Quintana M, Wörgötter F, Tetzlaff C, Fauth M. Modeling the shape of synaptic spines by their actin dynamics. Frontiers in Synaptic Neuroscience. 2020;12:9.
